# Evaluation of Cervical Cancer Screening in Japan: Challenges and Future Directions for Negative Intraepithelial Lesion or Malignancy/High-Risk Human Papillomavirus Positive Case Management

**DOI:** 10.3390/curroncol32060295

**Published:** 2025-05-23

**Authors:** Yasushi Umezaki, Asako Fukuda, Makiko Kurihara, Mariko Hashiguchi, Kaoru Okugawa, Masatoshi Yokoyama

**Affiliations:** Department of Obstetrics and Gynecology, Faculty of Medicine, Saga University, Saga 840-8502, Japan

**Keywords:** cervical cancer screening, HR-HPV, NILM/HR-HPV+, colposcopy, Japan healthcare policy

## Abstract

Cervical cancer screening is crucial for early detection and prevention. In Japan, women with negative intraepithelial lesion or malignancy (NILM) and high-risk human papillomavirus (HR-HPV) positivity are recommended retest for 12 months, rather than immediate colposcopy. International guidelines differ, and often prioritize early colposcopy for persistent HPV16/18 infections. This study evaluates Japan’s current screening approach, and identifies areas for improvement. A retrospective cohort study analyzed cervical cancer screening data from Saga Prefecture (2019–2021), assessing follow-up adherence, colposcopy referral rates, and CIN2+ and CIN3+ detection among NILM/HR-HPV+ cases. Among 27,789 individuals screened, 2248 (8.1%) were NILM/HR-HPV+. Follow-up adherence after 12 months was 54.4%. Of these, 132 with cytological abnormalities underwent colposcopy, revealing CIN2+ in 27.3% of cases. Additionally, 561 women with persistent NILM/HR-HPV+ underwent colposcopy, with CIN2+ in 7.6% and CIN3+ in 3.9% of cases. Japan’s current NILM/HR-HPV+ management strategy could delay the detection of high-grade cervical lesions. International guidelines favor earlier colposcopy referrals, particularly for HPV16/18+ cases. To improve cervical cancer prevention, Japan should consider a risk-based stratification model, enhance follow-up adherence, expand colposcopy access, and develop a national patient tracking system. Adopting primary HPV-based screening could attain the best global practices, facilitating earlier detection and reducing cervical cancer.

## 1. Introduction

Cervical cancer remains a major global health concern, with persistent infection by high-risk human papillomavirus (HR-HPV) recognized as the primary etiological factor. HPV16 and HPV18 are responsible for approximately 70% of cervical cancer cases worldwide [1]. The implementation of cervical cancer screening programs integrating cytology and HPV DNA testing has significantly reduced both the incidence and mortality of cervical cancer in many countries [2].

In Japan, cervical cancer screening has traditionally relied on cytology-based testing, and primary HPV testing has only recently been introduced as an optional co-testing method [3]. According to current Japanese guidelines, women who are negative for intraepithelial lesion or malignancy (NILM) but HR-HPV positive are recommended to undergo repeat testing after 12 months, without immediate colposcopy [3]. This conservative strategy is based on the assumption that transient HPV infections frequently clear spontaneously, and that cytological progression should serve as the main trigger for diagnostic colposcopy. However, this approach may delay the detection of high-grade cervical lesions, particularly in cases involving persistent HPV16/18 infections [4,5].

**Figure 1:**
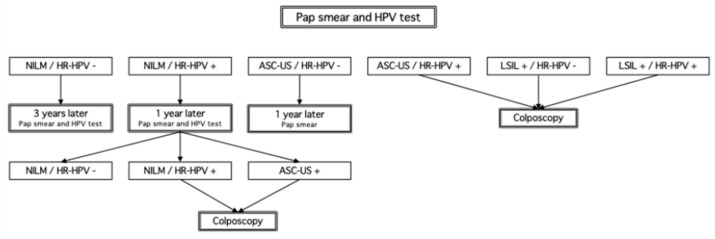
The cervical cancer screening and follow-up protocol currently implemented in Saga Prefecture. Under this protocol, women who test NILM/HR-HPV+ undergo repeat HPV and cytology testing after 12 months. If HR-HPV remains positive and cytological abnormalities develop, colposcopy is recommended. NILM: negative for intraepithelial lesion or malignancy; HR-HPV+: high-risk HPV positive; HR-HPV-: high-risk HPV negative; LSIL+: low-grade squamous intraepithelial lesion or worse; ASC-US+: atypical squamous cells of undetermined significance or worse.

Saga Prefecture has adopted a protocol aligned with these national recommendations, in which NILM/HR-HPV+ individuals undergo repeat testing after 12 months, and are referred to colposcopy only upon the emergence of cytological abnormalities (Figure 1). In contrast, international guidelines increasingly favor risk-based stratification models. The American Society for Colposcopy and Cervical Pathology (ASCCP) recommends immediate colposcopy for HPV16/18-positive women, regardless of cytology [4,5]. The United Kingdom’s National Health Service (NHS) Cervical Screening Programme advises colposcopy for women who remain HR-HPV positive for 2 consecutive years, even in the absence of cytological abnormalities [6]. In Australia, HPV-based primary screening was adopted in 2017, and HPV16/18 positivity alone is sufficient for immediate colposcopy referral [7,8].

Despite this global trend toward HPV genotype–driven triage, Japan has yet to fully implement such protocols nationwide. One barrier is the absence of a centralized national cervical screening registry. Unlike countries with integrated e-health infrastructures, Japan’s screening data are managed at the municipal level, often through paper-based systems or fragmented databases, which complicates longitudinal tracking and care coordination [3].

Another significant challenge is the lack of standardized triage algorithms specifically for women with NILM/HR-HPV+. While other countries stratify based on HPV genotype and consider additional molecular markers, Japanese guidelines treat all HR-HPV types similarly. This can lead to undertriage of high-risk cases. Mounting evidence suggests that HPV16/18-positive women, even with NILM cytology, face significantly elevated risks of developing CIN3+ and warrant immediate colposcopic evaluation [4,5].

Equally important is the low level of public awareness and health literacy regarding HPV and cervical cancer screening in Japan. Surveys have shown that many Japanese women are unaware of the need for follow-up after a positive HPV test, or mistakenly believe that the absence of symptoms equates to safety [9]. This informational gap contributes to low screening participation rates and poor follow-up adherence, particularly among younger women and those in rural or underserved areas.

Recent advancements in self-sampling for HPV testing may help address some of these barriers. Studies conducted both in Japan and internationally have demonstrated that self-collected samples offer comparable sensitivity to clinician-collected samples, especially when validated HPV assays are used [9]. Self-sampling could thus improve accessibility for women with childcare responsibilities, employment constraints, or geographic limitations. However, widespread implementation will require not only further clinical validation, but also regulatory approval and appropriate reimbursement frameworks.

This study aims to evaluate the clinical effectiveness and limitations of NILM/HR-HPV+ management in Japan, with a focus on Saga Prefecture as an early adopter of HPV co-testing. By analyzing real-world screening data, follow-up adherence, and referral patterns, we seek to highlight critical gaps and opportunities for aligning Japan’s screening protocols with international standards. A shift toward genotype-based triage, combined with better patient education and digital infrastructure, may significantly enhance Japan’s capacity to reduce the burden of cervical cancer.

## 2. Materials and Methods

### 2.1. Study Design

This study is a retrospective cohort analysis of cervical cancer screening data collected from Saga Prefecture, Japan, between January 2019 and December 2021. The primary objective was to evaluate the management of women with negative for intraepithelial lesion or malignancy (NILM) cytology and concurrent high-risk HPV (HR-HPV) positivity (NILM/HR-HPV+). Key outcomes included follow-up adherence, colposcopy referral rates, and histologically confirmed CIN2+ and CIN3+ detection rates. The results were contextualized through comparison with international management guidelines, including those of the American Society for Colposcopy and Cervical Pathology (ASCCP), the United Kingdom’s NHS Cervical Screening Programme, and the Australian National Cervical Screening Program.

### 2.2. Study Population

The study population was restricted to women aged 30 to 44 years, which represents a demographic with a high prevalence of HPV infection and growing concern for cervical cancer incidence in Japan. The target cohort consisted of individuals who underwent HPV co-testing (cytology + HPV DNA testing) as part of municipal or prefectural cervical cancer screening programs in Saga Prefecture. Women were excluded if they had a documented history of CIN2 or higher-grade lesions, had undergone colposcopy within the previous 12 months, or if their follow-up data were incomplete.

### 2.3. Data Collection and Variables

The primary data sources were standardized cervical cancer screening records managed by the Saga Prefecture Foundation for Health Promotion. Data were extracted from a centralized electronic registry that included HPV DNA test results (positive or negative status), cytological classifications, follow-up attendance, colposcopy referrals, and histopathological outcomes. Patient identifiers were anonymized using encrypted ID codes to protect privacy and ensure confidentiality.

HPV testing in this study was conducted using a clinically validated platform: the Cobas^®^ HPV test (Roche Molecular Systems, Mumbai, MH, USA). The Cobas HPV test detects high-risk HPV DNA, including HPV16, 18, 31, 33, 35, 39, 45, 51, 52, 56, 58, 59, 66, and 68. However, in this study, only binary HR-HPV test results (positive or negative) were available for analysis. Strain-specific genotyping information was not collected or analyzed, and thus no type-specific interpretation or correlation could be performed.

Cytology was performed using conventional Pap smears or liquid-based cytology. Cytological interpretation followed the Bethesda System 2014 criteria. Specimens were evaluated by licensed cytotechnologists with over 5 years of experience in cervical pathology. Indeterminate or borderline cases (e.g., ASC-US, LSIL) were reviewed by board-certified pathologists. Laboratories participated in the national external quality assurance program administered by the Japanese Society of Clinical Cytology.

Colposcopic examination was indicated for women with persistent HR-HPV infection and/or cytological abnormalities at follow-up. Colposcopy was conducted by certified gynecologic oncologists in designated regional hospitals. Biopsy was performed according to standard colposcopic criteria, including presence of acetowhite epithelium, vascular abnormalities (mosaicism, punctation), or suspicious lesions.

Follow-up adherence was assessed by the proportion of NILM/HR-HPV+ women who returned for repeat HPV and cytology testing at the 12-month interval, as per national guideline recommendations. Additionally, data were stratified by age group and geographic location (urban vs. rural municipalities) to assess regional disparities in follow-up and referral practices.

### 2.4. Statistical Analysis

Descriptive statistics were used to summarize demographic and clinical characteristics. Follow-up rates were calculated as the proportion of NILM/HR-HPV+ individuals who completed a 12-month reassessment. The colposcopy referral rate was defined as the percentage of individuals referred to colposcopy among those with persistent HPV positivity and/or cytological abnormalities. Detection rates of CIN2+ and CIN3+ were expressed as proportions among colposcopically evaluated individuals.

Due to the retrospective nature and categorical outcomes of the dataset, statistical analyses were limited to frequency distributions and percentage calculations. No inferential statistical tests (e.g., chi-square, logistic regression) were performed.

### 2.5. Ethical Considerations

This study was conducted in accordance with the ethical principles outlined in the Declaration of Helsinki and the Ethical Guidelines for Medical and Biological Research Involving Human Subjects in Japan. The study protocol was reviewed and approved by the Ethics Committee of Saga University Faculty of Medicine. Given the retrospective design and use of anonymized secondary data, individual informed consent was waived by the committee.

## 3. Results

A total of 27,789 individuals underwent cervical cancer screening in Saga Prefecture between 2019 and 2021. Among them, 2248 individuals (8.1%) were identified as NILM/HR-HPV-positive. The follow-up adherence rate after 12 months was 54.4%, indicating that nearly half of the affected individuals did not return for their scheduled follow-up examination.

**Figure 2:**
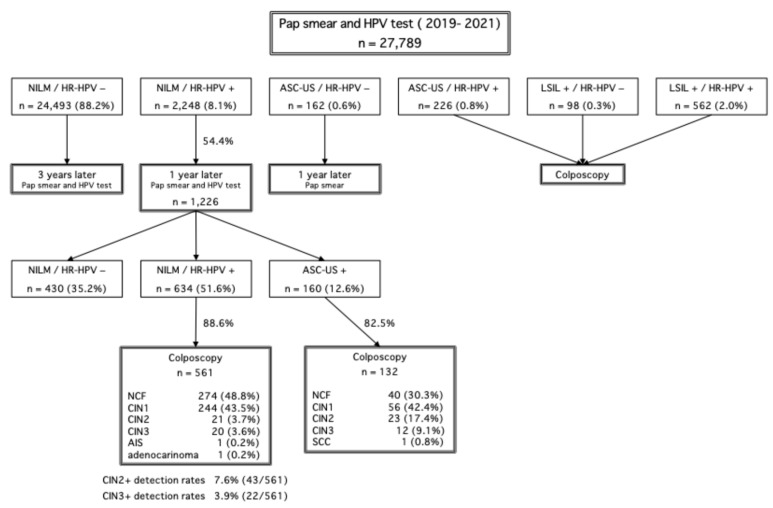
Summary of cervical cancer screening results in Saga Prefecture from 2019 to 2021. A total of 27,789 women underwent Pap smear and HR-HPV testing. Among them, 2248 women (8.1%) were classified as NILM/HR-HPV+, requiring further follow-up. The follow-up adherence rate after 1 year was 54.4%, with 1226 individuals undergoing repeat testing. NILM: negative for intraepithelial lesion or malignancy, HR-HPV+: high-risk HPV positive, HR-HPV-: high-risk HPV negative, LSIL+: low-grade squamous intraepithelial lesion or worse, ASC-US+: atypical squamous cells of undetermined significance or worse, NCF: normal colposcopic findings, CIN: cervical intraepithelial neoplasia, AIS: adenocarcinoma in situ, SCC: squamous cell carcinoma.

Among the 1226 individuals who attended follow-up, 132 cases with cytology results of ASC-US or higher underwent colposcopy. The colposcopy findings revealed that 30.3% of cases were negative for CIN (normal colposcopic findings [NCF]), 42.4% had CIN1, 17.4% had CIN2, 9.1% had CIN3, and 0.8% were diagnosed with squamous cell carcinoma. Additionally, among 561 individuals who underwent colposcopy due to persistent HR-HPV positivity for two consecutive years despite NILM cytology, 48.8% were negative for CIN (NCF), 43.5% had CIN1, 3.7% had CIN2, 3.6% had CIN3, 0.2% had adenocarcinoma in situ, and 0.2% had invasive adenocarcinoma. The CIN2+ detection rate in this group was 7.6%, while the CIN3+ detection rate was 3.9%. These colposcopy results are detailed in Figure 2.

## 4. Discussion

This study highlights the structural and clinical challenges associated with Japan’s current management of women with negative for intraepithelial lesion or malignancy (NILM) but high-risk HPV (HR-HPV) positivity, particularly when compared to internationally established guidelines. One of the core issues is that colposcopy referrals are contingent on the presence of cytological abnormalities. As a result, even women with persistent HR-HPV infection may not be referred for diagnostic colposcopy unless abnormal cytology eventually appears [3].

In many Japanese municipalities, if a woman remains NILM/HR-HPV+ after a 12-month follow-up, she is typically scheduled for another follow-up after an additional 12 months, without undergoing colposcopy [3]. This conservative algorithm may repeat indefinitely as long as cytological findings remain normal, despite ongoing viral persistence. The approach poses particular concern for HPV16/18-positive cases, which are associated with a substantially higher risk of CIN3+ lesions [4,5]. International consensus and large-scale cohort studies have demonstrated that the risk of CIN3+ in women who are HPV16/18-positive with normal cytology exceeds referral thresholds used in most Western guidelines [2].

Saga Prefecture represents a more progressive approach within Japan, wherein women with two consecutive NILM/HR-HPV+ results are referred for colposcopy. This strategy aligns more closely with evidence-based risk stratification models. However, in many other municipalities, including resource-limited and rural regions, such practices have not been adopted. As a result, a substantial proportion of at-risk women may remain undiagnosed or inadequately managed [3].

In contrast to Japan’s cytology-centered protocol, the ASCCP guidelines in the United States recommend immediate colposcopy for all HPV16/18-positive women, regardless of cytology results [4]. The United Kingdom’s NHS Cervical Screening Programme recommends colposcopy after two consecutive HR-HPV+ results, even in the absence of cytological abnormalities [6]. Similarly, Australia has adopted a fully HPV-based primary screening program, in which HPV16/18 positivity directly triggers colposcopy referral [7,8]. These countries have shifted toward a risk-based management framework, driven by evidence that HPV testing offers superior sensitivity and long-term negative predictive value for detecting high-grade cervical neoplasia [2].

Another critical concern revealed in this study is the suboptimal follow-up adherence. Among women with NILM/HR-HPV+ results in Saga Prefecture, only 54.4% returned for their 12-month follow-up test. This figure is consistent with previous findings in other regions of Japan. Low adherence undermines the effectiveness of screening and increases the risk of delayed diagnosis. Previous studies have demonstrated that targeted interventions—such as SMS reminders, telephone outreach, and digital health apps—can significantly improve follow-up rates [9].

One of the structural limitations in Japan is the absence of a centralized national cervical cancer screening registry. Without longitudinal tracking, it is difficult to ensure consistent follow-up, conduct program quality assurance, or analyze cumulative screening history over time. In contrast, countries such as the United Kingdom (through the Open Exeter system) and the Nordic countries operate integrated registries that support individualized risk assessment, timely referral, and continuous policy improvement [6,7]. Implementing a similar national database in Japan could bridge follow-up gaps and reduce regional disparities in clinical outcomes.

In addition, Japan faces notable inconsistencies in publicly funded support for cervical cancer screening. Municipal subsidies for HPV testing or colposcopy differ widely depending on local budget allocations, population size, and policy priorities. This inconsistency leads to unequal access to secondary screening and diagnostic services, particularly disadvantaging women in smaller municipalities or low-resource areas. Without standardized subsidy frameworks, such disparities may exacerbate existing socioeconomic inequities in cancer prevention outcomes [3,9].

Improving accessibility to colposcopy services is also crucial. In rural or underserved regions, the shortage of trained colposcopists and the lack of referral centers create logistical barriers that delay diagnosis. Establishing regional colposcopy hubs and training local providers could help mitigate these issues. Moreover, introducing national-level triage criteria based on HPV genotype and repeat positivity—rather than cytology alone—could improve diagnostic precision while optimizing healthcare resources.

### Limitations

This study focused on women aged 30 to 44 years, a demographic with high HPV prevalence, and was conducted with funding from Saga Prefecture. As the data were limited to this age group, different results may be obtained when including a broader age range. Particularly, in women over 50 years of age, HPV clearance rates are known to change, necessitating further investigation into age-specific management strategies.

In addition, although the HPV test platform used (Cobas^®^ HPV test) is capable of genotyping specific high-risk HPV strains, such as HPV16 and HPV18, the dataset available for this study included only binary HR-HPV results (positive or negative), without type-specific information. Therefore, we were unable to evaluate the distribution of oncogenic HPV strains, or assess the relationship between specific HPV types and colposcopy or follow-up outcomes. This limitation underscores the need for future studies incorporating genotype-stratified data to better inform risk-based screening policies.

Future research should explore screening policies across diverse age groups and incorporate type-specific HPV data to optimize management strategies.

## 5. Conclusions

This study highlights the need for revisions in Japan’s cervical cancer screening, particularly for NILM/HR-HPV+ cases. The current reliance on cytology for colposcopy referrals delays the detection of high-grade lesions, increasing the risk of missed diagnoses. Compared to international guidelines that prioritize earlier colposcopy, Japan’s approach may need adjustment to improve early detection.

Improving follow-up adherence and expanding colposcopy access are crucial to optimizing patient outcomes. Transitioning to a primary HPV-based screening model, as implemented in other countries, could enhance detection efficiency and reduce cervical cancer incidence. Further research is necessary to assess the feasibility and effectiveness of these proposed changes.

## Data Availability

The original contributions presented in this study are included in the article. Further inquiries can be directed to the corresponding author.

## Abbreviations

The following abbreviations are used in this manuscript:

NILM	intraepithelial lesion or malignancy
HR-HPV	high-risk human papillomavirus
ASCCP	American Society for Colposcopy and Cervical Pathology
CIN	cervical intraepithelial neoplasia
NCF	normal colposcopic findings
